# Heritable variation and lack of tradeoffs suggest adaptive capacity in *Acropora cervicornis* despite negative synergism under climate change scenarios

**DOI:** 10.1098/rspb.2021.0923

**Published:** 2021-10-13

**Authors:** Erinn M. Muller, Ashley M. Dungan, Wyatt C. Million, Katherine R. Eaton, Chelsea Petrik, Erich Bartels, Emily R. Hall, Carly D. Kenkel

**Affiliations:** ^1^ Mote Marine Laboratory, Sarasota, FL, USA; ^2^ School of BioSciences, The University of Melbourne, Parkville, Victoria, Australia; ^3^ Department of Biological Sciences, University of Southern California, Los Angeles, CA, USA; ^4^ Mote Marine Laboratory, International Center for Coral Reef Research and Restoration, Summerland Key, FL, USA

**Keywords:** *Acropora cervicornis*, global climate change, ocean acidification, synergistic effects, heritability, conferred resistance

## Abstract

Knowledge of multi-stressor interactions and the potential for tradeoffs among tolerance traits is essential for developing intervention strategies for the conservation and restoration of reef ecosystems in a changing climate. Thermal extremes and acidification are two major co-occurring stresses predicted to limit the recovery of vital Caribbean reef-building corals. Here, we conducted an aquarium-based experiment to quantify the effects of increased water temperatures and *p*CO_2_ individually and in concert on 12 genotypes of the endangered branching coral *Acropora cervicornis,* currently being reared and outplanted for large-scale coral restoration. Quantification of 12 host, symbiont and holobiont traits throughout the two-month-long experiment showed several synergistic negative effects, where the combined stress treatment often caused a greater reduction in physiological function than the individual stressors alone. However, we found significant genetic variation for most traits and positive trait correlations among treatments indicating an apparent lack of tradeoffs, suggesting that adaptive evolution will not be constrained. Our results suggest that it may be possible to incorporate climate-resistant coral genotypes into restoration and selective breeding programmes, potentially accelerating adaptation.

## 1. Introduction

Climate change poses myriad threats to planetary life. Reef-building coral and the ecosystems they support face some of the most immediate challenges as thermal stress-induced bleaching and subsequent mass mortality events are increasing in frequency and severity worldwide [1]. Although thermal stress poses the greatest threat to the long-term persistence of reefs, additional direct and indirect effects of climate change and other anthropogenic processes have also influenced the global decline of reefs [2,3]. An ever-increasing body of literature shows that coral are capable of adapting to thermal stress [4–6]. However, less is known about adaptive capacity in response to these other stressors (but see [7,8]), and the costs and/or tradeoffs of such adaptation also remains largely unresolved [9]. Understanding multi-stressor interactions and the potential for tradeoffs among tolerance traits will be essential for undertaking management interventions aimed at conserving and restoring reef ecosystems in the face of climate change.

Florida's Coral Reef (FCR) exemplifies the multi-stressor environment and patterns of reef decline predicted to occur on many reefs around the world as climate change progresses. Over the last 50 years, the state of FCR has changed drastically. In the 1970s and 1980s, many reefs were documented with 30–50% living coral cover, high fish diversity and significant structural integrity [10]. However, recent surveys indicate living coral cover has declined to only 5% of the benthic substrate [11], and further declines have occurred as a result of the most recent disease outbreak [12,13]. These significant losses are attributed to a combination of disease outbreaks in the dominant reef-builders, *Acropora* spp. [14,15], and increasing water temperatures, which have led to regional bleaching events [16,17]. FCR also experiences a combination of ocean and coastal acidification processes [18–20], which can impair coral growth and skeletal integrity [21–25]. Ocean acidification is the global decrease in oceanic pH as a result of the exponential increase of atmospheric carbon dioxide since the Industrial Revolution, which in turn leads to increases in dissolved CO_2_ within the oceans [26,27]. Studies have shown reefs of the Florida Keys have a net dissolution rate [18], are a net sink for CO_2_ [28] and have decreased aragonite saturation states [29], suggesting acidification may already be affecting coral reefs in this region [18].

The precipitous decline of the dominant reef-building species, *Acropora palmata* and *Acropora cervicornis*, has resulted in large-scale efforts to restore these species throughout FCR [30–32]. These efforts represent a significant investment of both monetary and human capital [33], and work in this area is only increasing. In fact, one of the largest coordinated coral restoration efforts planned in the world is being implemented throughout several ‘iconic’ reefs within the Florida Keys region. However, evidence suggests that current coral restoration efforts may not provide the solution for long-term recovery as high mortality rates occur within just a few years after outplanting, precluding ecosystem recovery [32,34]. Addressing the major threats that caused reef decline in the first place will be essential for the long-term success of these efforts. Moreover, additional interventions aimed at increasing the adaptive capacity of restored populations will probably also be key [35] as these novel approaches can provide critical stopgaps to allow species and ecosystems to persist while societal changes are enacted to curb global emissions.

The adaptive capacity of a population depends on genetically based variation in traits, or phenotypes, that selection can act upon [36]. The heritability of a trait is the proportion of phenotypic variation explained by genetic variation among individuals, which largely determines the magnitude and speed of phenotypes upon which selection can act [37]. Many studies have examined mean responses of corals to climate change stressors, yet comparatively few have measured individual-level variation and heritability of these responses in coral hosts [6,38–40], symbionts [41] or holobionts [41,42], and to date, these studies have largely focused on single phenotypic traits. In addition, traits are not independent and selection for one trait may result in unintended changes in other, correlated traits [43,44]. For example, a prior study showed that genotypes of *A. cervicornis* with higher initial growth rates ultimately lost more live tissue, reinforcing the potential for tradeoffs [45]. To date, the most comprehensive attempt to quantify tradeoffs among coral phenotypes remains a short-term laboratory experiment involving *A. millepora* where only positive correlations were detected [9]. Understanding potential tradeoffs associated with climate resistance will be essential to implement selective propagation and breeding as an intervention strategy.

With the loss of *A. cervicornis* throughout much of its range and the recent focus on outplanting tens of thousands of these corals each year to FCR, it is critical to understand phenotypic response and resistance of corals to major threats facing the reefs of Florida; threats that will probably persist for decades to come. In addition, there have been no studies to date quantifying potential broad-spectrum resistance or tradeoffs between heat tolerance and resistance to ocean acidification. Therefore, the objectives of the present study were to quantify: (i) the physiological response of *A. cervicornis* to chronically elevated temperature, ocean acidification conditions or the combination of the two; (ii) variation among genotypes in response to these threats; (iii) heritability of these phenotypic traits; and (iv) potential tradeoffs or conferred resistance to heat tolerance and ocean acidification.

## 2. Methods

### (a) Coral collections

On 11 July 2016, a total of 240 *A. cervicornis* fragments, each 5 cm long, were collected from Mote Marine Laboratory's offshore, *in situ*, coral nursery, located in the lower Florida Keys (24.56257° N, 81.40009° W). Twenty fragments with one apical polyp were collected haphazardly throughout the nursery from each of the 12 genotypes and transported back to Mote's Elizabeth Moore International Center for Coral Reef Research and Restoration in Summerland Key, Florida. Upon arrival, each coral fragment was glued onto a PVC cap using cyanoacrylate gel and placed within an 18.9 l aquarium tank. Tanks were held within two raceway tables at Mote's Climate and Acidification Ocean Simulator (CAOS) system. Each raceway held 10 tanks that were supplied with flowing seawater from individual spigots. Each tank held a single replicate of each genotype (i.e. 12 corals per tank).

### (b) Experimental design

The experimental design consisted of four treatment conditions referred to as follows. (i) Control: 704 ± 62 µatm *p*CO_2_, 27.1 ± 0.05°C; (ii) high temperature: 798 ± 62 µatm *p*CO_2_, 31.0 ± 0.04°C; (iii) high *p*CO_2_: 1225 ± 98 µatm *p*CO_2_, 27.0 ± 0.02°C; and (iv) combined: 1412 ± 90 µatm *p*CO_2_, 31.1 ± 0.05°C (electronic supplementary material, tables S1–S3). There were five replicates of each coral genotype per treatment. The goal was to cause significant sublethal stress to assess physiological responses after prolonged exposure to the four different environmental scenarios. All corals were initially maintained under normal, ambient temperatures at 30.35 ± 0.2°C to mimic conditions at the time of sampling. After the corals were acclimated to the CAOS system for a week, the treatment temperature and *p*CO_2_ conditions were reached by incremental changes to the parameters through time. To achieve treatment values, the temperature of the approximately 27°C temperature treatments were decreased −0.5°C d^−1^ (over the span of 7 days) and held at approximately 27°C. Simultaneously, the temperature of the high-temperature treatments was increased at 0.75°C d^−1^ (over the span of 2 days) and held at approximately 31°C. The high *p*CO_2_ treatments were decreased by 0.1 pH units per day (over the span of 4 days) by bubbling CO_2_ within the source water header tank. Although the target goal for *p*CO_2_ was approximately 450 µatm (average open ocean conditions), the low pH of the near-shore water source and the challenges associated with off-gassing CO_2_ prevented reaching this goal. As such, *p*CO_2_ was maintained at approximately 750 µatm within the control and high-temperature treatment (see electronic supplementary material, table S3 for treatment metrics). After reaching the treatment conditions, the corals remained within these conditions for two months. Temperature, salinity, dissolved oxygen, water turnover rate, photosynthetically active radiation, total pH, total alkalinity, *p*CO_2_, HCO_3_, CO_3_^2^ and the aragonite saturation state were measured to characterize the water quality throughout the experiment (see electronic supplementary material for detailed methodologies).

### (c) Phenotype measurements

We measured 12 phenotype traits throughout the experiment to assess differences in physiological responses among treatments and genotypes. Detailed methodologies for each metric are provided within the electronic supplementary material's phenotype measurements. Buoyant weight [46] was measured at the beginning and end of the experiment to quantify growth. To determine the status of the photochemical efficiency [47] of the algal symbionts, parameters including maximal quantum yield of PS II (Δ yield) and maximum electron transport rate (ΔETR_m_) were quantified by taking measurements prior to treatments and after two months of exposure. At the completion of the experiment, photosynthesis : respiration rate ratios were measured as a proxy for metabolic activity of the coral holobiont, and calcification rates were assessed using the alkalinity anomaly method as a secondary metric to quantify growth. Upon completion of these metrics, all coral samples were snap-frozen using liquid nitrogen until further processing at University of Southern California. Here, corals were processed for total soluble protein, prophenoloxidase (PPO), phenoloxidase (PO) and peroxidase (POX) as well as total chlorophyll (chl_Total_) concentrations and algal symbiont densities. The melanin synthesis cascade, a key component of innate immunity in invertebrates, is activated by the proteolysis of PPO to form active PO [48]. Reactive oxygen intermediates are generated as part of this cascade, which can be cytotoxic to pathogens as well as to the coral host, consequently, antioxidants such as POX are formed, presumably as a means of minimizing cytotoxicity to host cells [48]. *Acropora cervicornis* genets are known to vary in disease susceptibility [49] and immune competence; therefore, the activity of these three enzymes was quantified as a proxy of host immune function. Chlorophylls *a* and *c*2 (chl_Total_) are major components of the Symbiodiniaceae light-harvesting system and their loss, either through a reduction in the content per symbiont cell or the overall reduction of symbiont density is the manifestation of bleaching [50] while protein content is a proxy for host tissue biomass [51]. In all cases, corals within the control treatment were considered at a standard level of performance. Because potentially ‘beneficial’ metrics, such as significant growth or higher immune function under treatment scenarios were not observed, deviations from the control metrics were assumed to be physiological negative responses.

### (d) Statistical analyses

#### (i) Treatment effects

All statistical analyses were completed using R v. 4.0.3 [52]. A permutation multivariate analysis of variance (PERMANOVA) was used to determine whether the suite of physiological response variables significantly differed among treatments using the vegan package [53]. Data were log-transformed prior to analysis for standardization purposes. Pairwise PERMAVOVAs with a Bonferroni correction was used to identify which treatments differed from others. Linear mixed-effects models within the lmerTest package [54] were used for each dependent variable to determine whether there were differences among treatments, genotypes or interactions between the two fixed factors. Tank was included as a random variable within the model framework. *Post hoc* Tukey HSD tests were applied to determine which treatment was significantly different from others when differences were detected in the linear mixed-effects model.

#### (ii) Heritability

To assess broad-sense heritability (*H^2^*), we used a Bayesian modelling approach similar to Kenkel *et al*. [39]. The heritability models were fitted using the package MCMCglmm [48]. The model included temperature and *p*CO_2_ treatment and their interaction as fixed effects, and scalar random effects of tank and genotype (to capture broad-sense heritability). To assess the proportion of the variance associated with broad-sense heritability, we divided the variance due to genotype effects by the sum of the variance from all random factors. All MCMC chains were run for 50 000 iterations, discarding the first 10 000 as a burn-in period, after which the chain was sampled every 20 iterations resulting in 2000 samples of each parameter value. The mean and quantiles of the broad-sense heritability were then calculated for the sampled values of the random genotype effect.

#### (iii) Tradeoffs/broad-spectrum resistance

To determine whether there were correlations among traits measured, we conducted a correlation matrix analysis among traits for each treatment. Although some traits indeed correlated, the treatment responses were often unique to each metric. Therefore, the average value for each trait of every genotype was calculated under the four different treatment conditions. The average genotypic response was then processed through a correlation matrix for each variable to assess whether there were tradeoffs with (significant negative correlation values) or broad-spectrum resistance to (significant positive correlation values) exposure to prolonged high temperature or ocean acidification conditions or the combination of these two stressors. The function corrplot [55] was used to create the correlation matrix plots and the function rcorr within the package ‘Hmisc’ [56] was used to calculate the correlative *p*-values for genotypes among treatments. This process was used to test for correlations among traits measured and to explore explicit tradeoffs.

## 3. Results

### (a) Population-level response to individual and combined stressors

**Figure 1.**
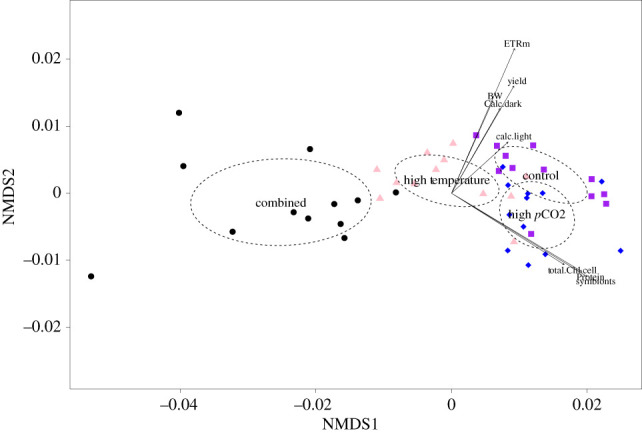
Non-metric multidimensional scaling ordination plot showing the spatial orientation of the average response of each coral genotype after two months of treatment. Purple squares, control; blue diamonds, high *p*CO_2_; light pink triangles, high temperature; black circles, combined treatments. Arrows depict the eigen value of each phenotype measured within the study with a *p*-value of <0.01; stress = 0.05. (Online version in colour.)

Out of the 240 fragments within the experiment, only two died (one in the control and one in the high *p*CO_2_ treatment), providing a robust dataset of physiological traits. The PERMANOVA, which included all phenotypes for each genotype, showed significant differences among treatments (*F*_3,47_ = 36.13, *R*^2^ = 0.711, *p* = 0.001; figure 1). *Post hoc* analyses showed the combined treatment was significantly different from all other treatment conditions (control versus combined: *p* = 0.006, high temperature versus combined: *p* = 0.006, high *p*CO_2_ versus combined: *p* = 0.006). Additionally, the control treatment was significantly different from the high-temperature treatment (*p* = 0.006) and the high-temperature treatment was also significantly different from the high *p*CO_2_ treatment (*p* = 0.006). Interestingly, the high *p*CO_2_ treatment was not significantly different from the control treatment (*p* = 0.060).

The vectors associated with the significant parameters that contributed to the prediction of ordination among treatments orient in two main directions along NMDS2 (*y*-axis). There is a cluster of responses including total soluble protein, algal symbiont concentrations and chl_Total_, which are all similar in magnitude and appear to characterize the high *p*CO_2_ treatment. The second cluster characterizes the control treatments with vectors including calcification metrics, buoyant weight and the photochemical parameters. NMDS1 (*x*-axis) shows that corals within the combined conditions were the farthest away from the correlative vectors, suggesting reductions in all parameters measured characterizes this treatment condition. Furthermore, P : R as well as the full suite of host immune proteins (PPO, PO and POX) were not significant indicators for ordination.

### (b) Holobiont response

#### (i) Buoyant weight

**Figure 2.**
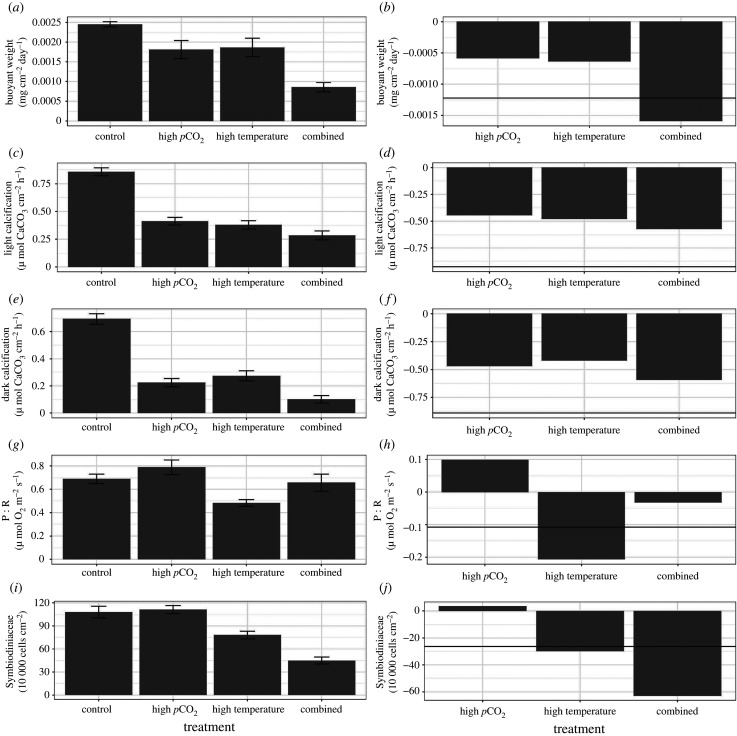
Average responses of holobiont traits of *A. cervicornis* corals after two months of exposure to four different temperatures treatments (left panel). Error bars represent standard error of the mean. The right panel shows the relative difference of each treatment condition compared with the response of corals within control treatments. The horizontal line depicts the additive response of the high-*p*CO_2_ and high-temperature treatments. Exceeding the line in the combined scenario suggests synergistic effects, a value similar to the line depicts additive effects and a value less than the line suggests antagonistic effects.

There were significant differences in buoyant weight among treatments (*F*_3,16_ = 5.94, *p* = 0.006; figure 2*a*). However, *post hoc* comparison showed the individual stressors were not statistically different from controls (control versus high *p*CO_2_: *p* = 0.066, control versus high temperature: *p* = 0.107). When high temperature and high *p*CO_2_ conditions were combined (i.e. combined conditions) buoyant weight significantly differed from all others (*p* < 0.001 for all comparisons). In fact, the buoyant weight within the combined treatment showed negative synergistic effects, where the additive effects of both high water temperature and high *p*CO_2_ (horizontal line in figure 2*b*) were far exceeded. Interestingly, there were no significant differences in buoyant weight among genotypes (*F*_11,172_ = 1.39, *p* = 0.18), and no significant interaction between treatment and genotype (*F*_33,172_ = 1.12, *p* = 0.42).

#### (ii) Calcification

Both calcification rates in the light and the dark declined significantly for corals within the high *p*CO_2_, high temperature and combined treatment scenarios (light: *F*_3,16_ = 30.36, *p* < 0.001, figure 2*c*; dark: *F*_3,16_ = 20.67, *p* < 0.001, figure 2*e*). All three treatment conditions showed significantly reduced calcification rates by over 50% when compared with controls (*p* < 0.001 for all comparisons). There were significant differences detected among genotypes for the light calcification metric (*F*_11,173_ = 1.94, *p* = 0.04) and a significant interaction between treatments and genotypes were detected (*F*_33,172_ = 1.65, *p* = 0.02) suggesting that genotypes differed in response to the four environmental conditions. However, there were no differences among genotypes for dark calcification rates (*F*_11,173_ = 1.77, *p* = 0.06) and there was no significant interaction between treatment conditions and genotypes (*F*_33,173_ = 0.63, *p* = 0.94). Interestingly, there was an antagonistic interaction between high temperature and high *p*CO_2_ conditions where the combined treatment had less of an effect compared with the additive response of the other two treatments independently (figure 2*d,f*).

#### (iii) Photosynthesis/respiration

There were no significant treatment effects on the P : R ratio (*F*_3,16_ = 1.002, *p* = 0.417); however, there was a significant difference among genotypes (*F*_11,170_ = 2.60, *p* = 0.004, figure 2*g*). There was no interaction detected between treatment conditions and genotypes (*F*_11,170_ = 1.331, *p* = 0.124). Similar to the calcification rate metrics, there was an antagonistic response within the combined treatment where the decreasing P : R ratio under high temperatures and the increasing P : R ratio under high *p*CO_2_ had opposing effects when combined (figure 2*h*).

#### (iv) Symbiodiniaceae concentrations

There were significant differences in algal symbiont concentrations (Symbiodiniaceae cm^−2^) within corals among treatments (*F*_3,19_ = 4.94, *p* = 0.01) with all treatments significantly differing from each other (*p* < 0.001 for all comparisons) except the high *p*CO2 and the control treatments (*p* = 0.99, figure 2*i*). There were also significant differences detected among genotypes (*F*_11,171_ = 3.02, *p* = 0.001), but significant interactions were not detected (*F*_33,171_ = 1.27, *p* = 0.163). Symbiodiniaceae concentrations showed a high level of synergy under combined treatment conditions with a large reduction in the number of algal cells compared with either treatment independently or the additive response (figure 2*j*).

### (c) Host response

#### (i) Soluble host protein

**Figure 3.**
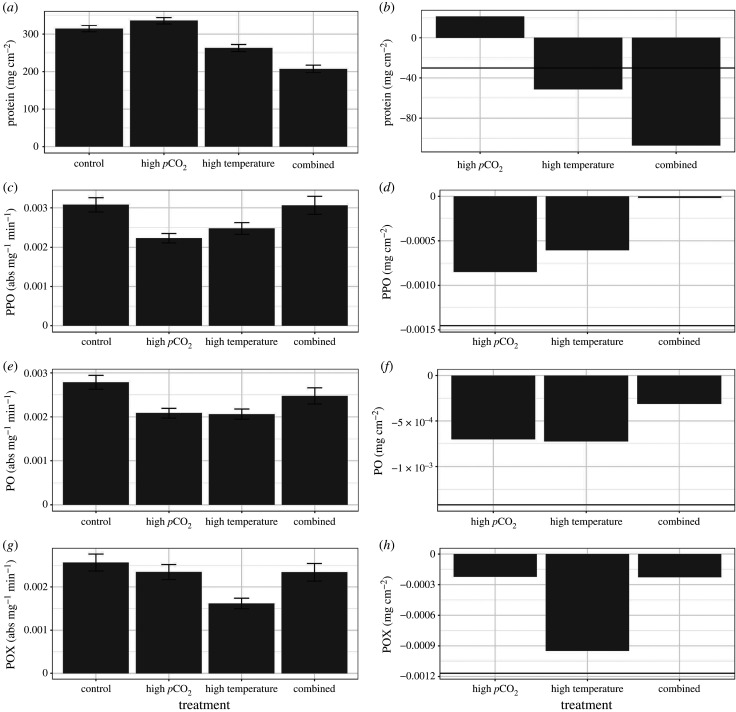
Average responses of host traits of *A. cervicornis* corals after two months of exposure to four different temperatures treatments (left panel). Error bars represent standard error of the mean. The right panel shows the relative difference of each treatment condition compared with the response of corals within control treatments. The horizontal line depicts the additive response of the high-*p*CO_2_ and high-temperature treatments. Exceeding the line in the combined scenario suggests synergistic effects, a value similar to the line depicts additive effects and a value less than the line suggests antagonistic effects.

The concentration of soluble protein (mg cm^−2^) of the coral host significantly differed among treatments (*F*_3,17_ = 8.71, *p* < 0.001, figure 3*a*) and by genotype (*F*_11,171_ = 2.36, *p* = 0.009), but there was no significant interaction between the two variables (*F*_33,171_ = 0.59, *p* = 0.96). The *post hoc* analysis showed that all treatments differed from each other (*p* < 0.001) except the high *p*CO_2_ treatment compared with the control treatment (*p* = 0.35). There was a synergistic response with a greater reduction in host protein concentration under combined treatment conditions than the additive null expectation for these responses (figure 3*b*).

#### (ii) Prophenoloxidase, phenoloxidase, peroxidase

There were no significant differences detected among treatments for prophenoloxidase (PPO), phenoloxidase (PO), or peroxidase (POX) (PPO: *F*_3,22_ = 0.531, *p* = 0.67, PO: *F*_3,20_ = 1.69, *p* = 0.20, POX: *F*_3,18_ = 2.62, *p* = 0.08, figure 3*c,e,g*). There were lower concentrations of these immune proteins in corals within the high-temperature treatment and the high *p*CO_2_ treatments alone, although these were not significantly different from controls after accounting for genotype variation and tank effects. Interestingly, corals within the combined conditions showed immune proteins comparable to corals within the control treatment conditions, suggesting that the combined variables of high *p*CO_2_ with high temperature had slight antagonistic effects on the immune protein response. There were significant differences among genotypes for all of the immune protein parameters (PPO: *F*_11,175_ = 5.21, *p* < 0.001, PO: *F*_11,177_ = 4.32, *p* < 0.001, POX: *F*_11,177_ = 4.69, *p* < 0.001), but no significant interactions were detected (POX: *F*_33,177_ = 1.50, *p* = 0.05; PO: *F*_33,177_ = 1.24, *p* = 0.187) except for PPO (F_(33,177)_ = 0.1.61, *p* = 0.03).

### (d) Symbiont response

#### (i) Maximum photochemical yield

**Figure 4.**
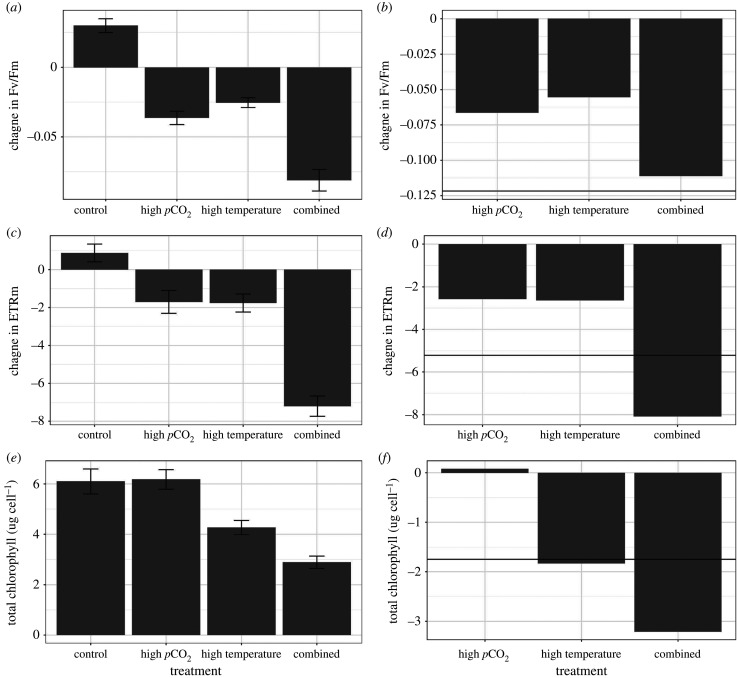
Average responses of algal symbiont traits of *A. cervicornis* corals after two months of exposure to four different temperatures treatments (left panel). Error bars represent standard error of the mean. The right panel shows the relative difference of each treatment condition compared with the response of corals within control treatments. The horizontal line depicts the additive response of the high-*p*CO_2_ and high-temperature treatments. Exceeding the line in the combined scenario suggests synergistic effects, a value similar to the line depicts additive effects and a value less than the line suggests antagonistic effects.

There were significant differences in yield among treatments (*F*_(3,16)_ = 16.07, *p* < 0.001; figure 4*a*) with all three treatment conditions showing a significant reduction in yield compared with control conditions (*p* < 0.001 for all comparisons). The high *p*CO_2_ and the high-temperature treatments did not differ (*p* = 0.43). There was a significant difference in yield among genotypes (*F*_(11,174)_ = 4.08, *p* < 0.001) as well as an interaction between treatments and genotypes (*F*_(33,174)_ = 2.44, *p* < 0.001). There was a slight antagonistic, or potentially simply an additive, response of yield when corals were exposed to the combined treatment conditions, where the reduction closely approximated the additive response of the high temperature and high *p*CO_2_ conditions (figure 4*b*).

#### (ii) Electron transport rate

The maximum electron transport rate (ETRm) significantly differed among treatments (*F*_3,16_ = 24.55, *p* < 0.001; figure 4*c*) with all treatments showing a significant reduction in ETRm compared with the corals in the control treatments (*p* < 0.001). The corals in the combined treatment showed a sevenfold reduction in ETRm compared with controls while high *p*CO_2_ and high-temperature treatments showed a threefold reduction. The ETRm of corals within the high *p*CO_2_ and high-temperature treatments did not significantly differ from each other (*p* = 0.99). There were significant differences among genotypes (*F*_3,175_ = 3.95, *p* < 0.001), but the interaction between treatments and genotypes was not significant (*F*_33,175_ = 1.20, *p* = 0.22). A large synergistic response was quantified for ETRm under the combined treatment conditions compared with the high temperature and high *p*CO_2_ treatment conditions (figure 4*d*).

#### (iii) Total chlorophyll concentrations

There were significant differences in chl_Total_ concentrations within corals among treatments (chl_Total_: *F*_3,16_ = 14.38, *p* < 0.001; figure 4*e*), with all treatments significantly differing from each other (*p* < 0.03 for all comparisons) except the high *p*CO_2_ compared with the control treatment (*p* = 0.99). There were also significant differences detected among genotypes (*F*_11,120_ = 5.69, *p* < 0.001), but significant interactions were not detected (*F*_33,120_ = 0.97, *p* = 0.53). The chl_Total_ concentrations showed a synergistic response under the combined treatment conditions with the reduction in chl_Total_ far exceeding the high-temperature treatment reductions (figure 4*f*). Interestingly, there was no effect of the high *p*CO_2_ conditions alone, but this treatment combined with high temperatures caused much greater reductions in chl_Total_.

#### (iv) Broad-sense heritability

**Figure 5.**
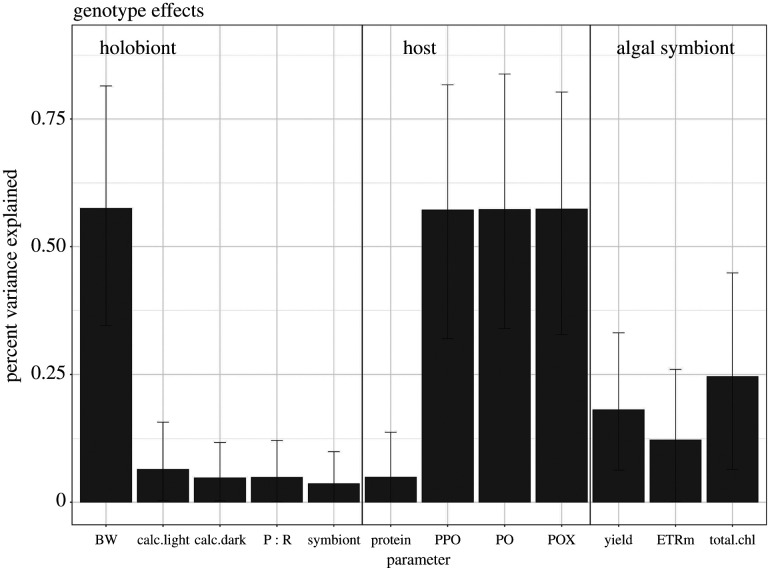
Average variance of each phenotype explained by genotype effects after integrating the treatment responses within the model. Error bars represent the 95% confidence interval of the mean.

The heritability analysis showed that approximately 57% of the variance of the major immune protein concentrations within the corals tested (PPO, PO and POX) was explained by genotype (figure 5). A similar level of heritability was quantified for buoyant weight (approx. 57% of the variance). All other parameters showed approximately 20% or less of the variance was explained by the coral genotype. Separating the heritability model by treatment conditions overall reduced the ability to explain traits due to shared inheritance (electronic supplementary material, figure S1).

#### (v) Tradeoffs versus broad-spectrum resistance

**Figure 6.**
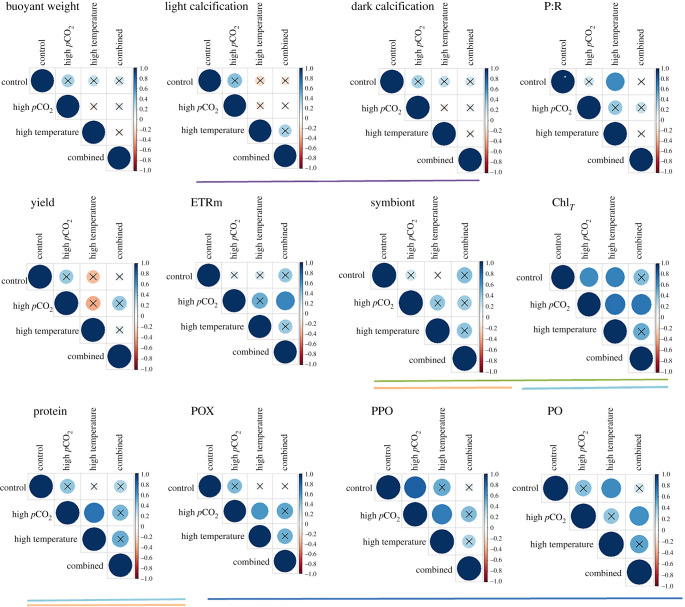
Results of the correlation matrix analyses conducted on the average response of each genotype for each parameter under the four treatment scenarios (control, high temperature, high *p*CO_2_ and combined). Colour and size denote the magnitude and direction of the correlation coefficients. Black ‘×’s denote non-significant associations in genotypic response when applying a Pearson correlation test. Horizontal lines of similar colours denote phenotypic metrics that correlate with each other. (Online version in colour.)

The correlation analyses among traits measured showed some correlations (electronic supplementary material, figure S2). Particularly, PPO, PO and POX positively correlated with each other within each treatment group. Additionally, the two metrics of calcification (light and dark) were positively correlated. Finally, algal symbiont concentrations positively correlated with chl_Total_ and host protein concentrations (all but the control treatment); chl_Total_ positively correlated with host protein concentrations as well (all but the high-*p*CO_2_ treatment). The majority of correlations among response variables showed positive associations, suggesting that responses of individual genotypes followed a similar pattern among treatments (i.e. when values increased under one treatment they also increased under the comparative treatment, or values similarly declined; figure 6). Statistically significant associations were always positive and were detected for P : R, ETRm, chl_Total_, protein, PPO, PO and POX concentrations (figure 6). Significant positive associations between three key comparisons essential for detecting tradeoffs or conferred broad-spectrum resistance within future ocean conditions (high temperature versus high *p*CO_2_, high temperature versus combined and high *p*CO_2_ versus combined) were not consistently observed for any particular trait measured but were observed at least once for ETRm, chl_Total_, host protein, PPO, PO and POX. Interestingly, the most commonly measured phenotypes used for assessing responses to threats such as thermal stress and ocean acidification showed no significant correlations (BW, calcification and yield). Although BW, calcification and yield all showed some negative associations, the correlations were weak and not significant.

## 4. Discussion

### (a) Synergistic response to climate change stressors

Climate change is negatively impacting reef-building corals [57], but the relative importance of individual stressors and the potential for non-additive interactions among them remains unclear [58]. We adopt the definition of synergy as an interaction in which the physiological response to the combined stress treatment is greater than the threshold calculated from each stressor in isolation [59]. In *A. cervicornis*, several of the physiological responses to combined high temperature and *p*CO_2_ treatment appeared synergistic (figures 2–4), which is largely in contrast with results from other studies on tropical corals [22,60–63]. Treatments within the present study, although constantly maintained, differed from target values due to the complexity associated with the experimental design and system limitations. Additionally, the interplay between water temperature and dissolved gases (negative association with dissolved oxygen and positive association with *p*CO_2_) prevented maintaining the same water conditions within the four treatment scenarios. These methodological limitations may have influenced phenotypic responses associated with treatment exposure within the present study.

Another factor potentially contributing to the synergistic responses detected in our study may be the choice of phenotypic traits measured. Buoyant weight and algal symbiont cell density, which reflect cumulative change over the entire two-month exposure, show synergistic negative impacts, whereas endpoint measures of calcification and P : R ratios exhibited multiplicative or additive effects. This finding is in contrast with a similar study where combined high temperature and *p*CO_2_ treatment resulted in additive declines on growth rate and symbiont density of *A. cervicornis* [60]. Other studies have also shown negative responses from elevated temperatures and *p*CO_2_, but no synergistic effects [61,62,64]. Synergistic negative impacts on calcification were observed in *Stylophora pistillata* following exposure to elevated *p*CO_2_ and temperature in combination, but symbiont cell densities actually increased [65]; calcification rate (µmol CaCO_3_ cm^−2^ h^−1^), on the other hand, was reduced by greater than 50% within all treatment conditions.

Host-specific traits also varied in their responses, with protein content showing a synergistic negative impact, whereas the activity of immune enzymes showed antagonistic effects. Although protein content is an endpoint measure, other studies have shown that protein content tracks holobiont growth over longer experimental durations [39] suggesting that this trait may be reflective of cumulative stress.

Cumulative measurements of the symbiotic algae were also assessed. While an antagonistic effect, closely approximating an additive response, was observed for yield, synergistic effects were observed for both ETRm and chl_Total_. Yield is an indicator of photosynthetic performance as it quantifies photons from photosynthetically active radiation used for photosynthesis within PSII of the chloroplasts within the Symbiodiniaceae [66]. However, ETRm, which represents electron flow beyond photosystem II, can be even more sensitive to thermal stress compared with yield [67]. The effect of combined conditions on ETRm and chl_Total_ showed synergistic effects with an approximate threefold reduction when stressors were combined compared with coral exposed to each stressor in isolation.

### (b) Heritable trait variation

We found significant differences in the phenotypic trait response to climate change stressors indicating high physiological variability among the genotypes tested, consistent with prior studies in *A. cervicornis* and other acroporids [9,42,68]. Importantly, a significant portion of this variation could be explained by coral genotype, reflective of broad-sense heritability (*H^2^*) for one trait (BW) specific to the holobiont. Although it is important to note that variance attributable to additive genetic effects, or narrow-sense heritability, is what determines the response to selection, *H*^2^ is a reasonable approximation of adaptive potential in long-lived, clonal organisms, such as corals [41] as it reflects the summed contribution of genetic, epigenetic and maternal effects in generating phenotypic variation [37]. Similar to Császár *et al*. [41], our results show high heritability of buoyant weight suggesting evolutionary potential for this trait across species. Kenkel *et al*. [39] report *H^2^* for buoyant weight and total linear extension of *A. cervicornis* from *in situ* nursery range from 0.25 to 0.28 [42]. Our comparatively higher *H^2^* estimate could be the result of a different sample population or different experimental conditions [69]. Importantly, our result reinforces the conclusion that growth is heritable in *A. cervicornis*. As growth rate is key for restoring the ecological function of reefs, this finding suggests that the propagation of high-growth rate genets in restoration populations will not alter growth rate in future temperature and acidification conditions, although additional *H^2^* estimates for growth in response to other local and global stressors are needed. Moreover, these coral genets were reared in a common garden nursery for a minimum of 5 years and are dominated by a common symbiont, *Symbiodinium fitti* [49], reducing potential variation among genets due to origin effects and symbiont type that can confound heritability estimates.

Unlike the findings of Császár *et al*. [41] who report low *H^2^* estimates for coral antioxidant expression, three of our highest *H^2^* estimates were detected for the activity of an antioxidant (POX) and a key component of the innate immune response (PPO and PO). It is possible that a difference in selective regime can explain this pattern, as heritability can increase in unfavourable conditions [70], although the opposite pattern is possible [71], and Caribbean coral reefs experience higher prevalence of disease than Indo-Pacific reefs [72]. Prior work has shown that there is natural variation in disease resistance among *A. cervicornis* genets [49,73], with proportionally more disease-resistant genets in Florida compared with Panama [49]. Decades of disease outbreaks and high heritability of immune activity could underpin this observation of evolutionary potential.

### (c) Lack of tradeoffs suggests broad-spectrum resistance

Heritable variation in climate resistance traits is essential for adaptive evolution; however, the rate of evolution will also be determined by tradeoffs in the response to individual stressors. Similar to the findings of Wright *et al*. [9], we failed to find significant tradeoffs in the response to temperature and acidification, in spite of several synergistic negative responses to combined stressors. Importantly, we find no significant negative correlations for any of the measured metrics. P : R, ETRm, chl_Total_, total soluble protein and immune protein (PPO, PO, POX) concentrations all showed significantly positive correlated responses to at least some of the treatment exposures, indicating that genets that perform well under one stressor will also likely perform well under another. Although this finding is trait-dependent, as calcification metrics (light and dark, BW) and photochemical parameters (yield and ETRm) showed no significant negative correlations, particularly under combined treatment conditions, these results are highly encouraging for practitioners aiming to increase the adaptive capacity of their restoration stock.

## 5. Conclusion

Development of intervention strategies for the conservation and restoration of reef ecosystems under climate change necessitates an understanding of multi-stressor interactions and the potential for tradeoffs among tolerance traits. This information is imperative for coral species already undergoing captive rearing and restoration, like *A. cervicornis* in the Caribbean. Our results suggest that this species will suffer significant negative effects from increasing thermal stress and ocean acidification, and that these two threats occurring together will often have synergistic effects on organismal physiology. However, significant variation in phenotypic responses among genotypes and positive trait heritability indicates that adaptive diversity is already present within restoration coral stock. In addition, our results also suggest that there is some level of broad-scale and conferred resistance to major global threats among genotypes as no significant tradeoffs were detected among a suite of holobiont, host and symbiont traits in response to our treatments. While reduction of carbon emissions is of paramount importance given the synergistic negative impacts observed here, taken together, our findings support the potential for captive rearing programmes to increase the resistance of restored populations of this key Caribbean reef-builder to temperature and acidification stress.

## Data accessibility

Data are available from the Dryad Digital Repository: https://doi.org/10.5061/dryad.mcvdnck1w [74]. The data are provided in electronic supplementary material [75].
