# Supplementary material for: Heritable variation and lack of tradeoffs suggest adaptive capacity in Acropora cervicornis despite negative synergism under climate change scenarios
Source: Proc Biol Sci. 2021 Oct 13;288(1960):20210923. doi: 10.1098/rspb.2021.0923 (PMC8511747; doi:10.1098/rspb.2021.0923)
Supplement: Supplementary file 1 [file rspb20210923_review_history.pdf]

THE ROYAL SOCIETY
PUBLISHING

PROCEEDINGS B

Heritable variation and lack of tradeoffs suggest adaptive capacity in *Acropora cervicornis* despite negative synergism under climate change scenarios

Erinn M. Muller, Ashley M. Dungan, Wyatt C. Million, Katherine R. Eaton, Chelsea Petrik, Erich Bartels, Emily R. Hall and Carly D. Kenkel

Article citation details
Proc. R. Soc. B **288**: 20210923.
http://dx.doi.org/10.1098/rspb.2021.0923

Review timeline
Original submission: 20 April 2021
Revised submission: 11 August 2021
Final acceptance: 21 September 2021

Note: Reports are unedited and appear as submitted by the referee. The review history appears in chronological order.

Review History

RSPB-2021-0923.R0 (Original submission)

Review form: Reviewer 1

Recommendation
Accept with minor revision (please list in comments)

Scientific importance: Is the manuscript an original and important contribution to its field?
Excellent

General interest: Is the paper of sufficient general interest?
Good

Quality of the paper: Is the overall quality of the paper suitable?
Good

Is the length of the paper justified?
Yes

Reports © 2021 The Reviewers; Decision Letters © 2021 The Reviewers and Editors; Responses © 2021 The Reviewers, Editors and Authors. Published by the Royal Society under the terms of the Creative Commons Attribution License http://creativecommons.org/licenses/by/4.0/, which permits unrestricted use, provided the original author and source are credited

Should the paper be seen by a specialist statistical reviewer?
No

Do you have any concerns about statistical analyses in this paper? If so, please specify them explicitly in your report.
No

It is a condition of publication that authors make their supporting data, code and materials available - either as supplementary material or hosted in an external repository. Please rate, if applicable, the supporting data on the following criteria.

Is it accessible?
Yes

Is it clear?
No

Is it adequate?
No

Do you have any ethical concerns with this paper?
No

Comments to the Author
This study tracks the performance of 12 coral genets that are currently used in restoration over two months in elevated temp and pCO2 lab treatments. Unlike many studies of this type, the authors track a large number of physiological trait responses (11 parameters, related to host, symbionts, and holobiont) to the climate change stressors with a relatively large number of genets. The results thus underscore that different traits show both different patterns of response to the stressors and different patterns of genotypic responsiveness (hence expected adaptability). The apparent lack of tradeoffs is particularly encouraging, and more convincing in this paper than others I am familiar with that have tried to examine this question. The paper is quite well-written and the data is clearly presented. I have primarily minor suggestions for clarification (and one slight correction).
My one substantive comment relates to the treatment conditions and the conclusions of widespread synergistic effects. However, it appears from Table 1 that the treatment levels in the Combined treatment were higher (and for temp considerably higher and more variable) than in the respective single stressor treatments. For temp this was 1.4oC higher (and it seems that a 1.2 degree standard error means it got substantially above 32.5 in this combined treatment for at least a portion of the time) which seems substantial to me. Could this increment in heat stress not account for some of the larger effect sizes that you attribute as synergism? This synergy of stressors is a major conclusion/discussion point which requires better justification given this apparent discrepancy in the treatment levels.

Minor points:
-Question: is there a reason the authors use the term 'Florida's Coral Reef' rather than the long established 'Florida Reef Tract'?
Ln 50-52: I found this a rather strong statement regarding Florida's reef currently (i.e. present tense) experiencing effects of acidification impairing growth and skeletal integrity and indeed, none of the cited references pertain to FCR. The Florida-related reference for reef dissolution in the following sentence does not distinguish effects of acidification from the simple fact that Floridas reefs simply have extremely low abundance of calcifying corals!
Ln 62-63: I am also not sure the statement that Floridas 'iconic reefs' restoration project is the 'largest . . planned in the world'. It is massive, but there are certainly similar planning efforts underway (if not as advanced as the Florida effort) for Australia's GBR no?
Ln 79: consider revision to 'phenotypes upon which selection can act'

Experimental design description: I know it is common practice to refer to certain treatments levels as 'ambient'. However, this the description for this paper, it is explicitly clear that the 'ambient' temperature treatment is not as it involved shifting from 30.35 at which the corals were collected to 27! If 27 is not the ambient temp, don't call it that. Just call it 27.5! I am not sure what the actual 'ambient' pCO2 level is . . . but please consider if the 'control' pCO2 level you are using is really 'ambient'
Ln122-124: It would be nice to include a sentence regarding how pCO2 was manipulated in this study (presumably by bubbling CO2 into the system water?)
Ln 144-146: I would have appreciated a sentence explaining why the PPO, PO, and POX are relevant or of interest.
Ln 171-175: My interpretation of this correlation analysis is that there is an unstated assumption that all of the parameters/traits measured are 'beneficial' in some sense, such that higher levels always represent a better status for the organism. Am I off base here? I am not completely convinced that this assumption makes sense (e.g., is higher symbiodinium cell density or higher protein content always better?). I guess I think the rationale for this analysis could use a better explanation (as I feel I am not understanding the logic).
Ln 199: Should this word be 'indicators'?
Ln 225 and Ln 260: these instances should be effect (not affect)
Ln 233: should be 'combined'
Ln 268: reconsider the term 'negative decline' – this sounds like a double negative that would be positive
Ln 350: delete one more
Figs 2-4: I like the way these figs are presented (very clear). However, I think the legend for Fig 4 should say 'symbionts' rather than 'host'
General for all figs: I imagine this is just an artifact of the proof (i.e. not the authors' doing), but the fonts are WAY too small on all the figure renderings in the review copy.

Review form: Reviewer 2

Recommendation
Major revision is needed (please make suggestions in comments)

Scientific importance: Is the manuscript an original and important contribution to its field?
Excellent

General interest: Is the paper of sufficient general interest?
Good

Quality of the paper: Is the overall quality of the paper suitable?
Good

Is the length of the paper justified?
Yes

Should the paper be seen by a specialist statistical reviewer?
No

Do you have any concerns about statistical analyses in this paper? If so, please specify them explicitly in your report.
No

It is a condition of publication that authors make their supporting data, code and materials available - either as supplementary material or hosted in an external repository. Please rate, if applicable, the supporting data on the following criteria.

Is it accessible?
Yes

Is it clear?
No

Is it adequate?
Yes

Do you have any ethical concerns with this paper?
No

Comments to the Author
I like this paper. It is novel and timely, and the experimental design and data analysis are rigorous and robust. The papers is clear and concise, very well written overall.

My issue with the paper is a conceptual one with regard to the choice of traits measured and tested. The conclusions of the paper are that tradeoffs between traits are not likely to impact survival of coral fragments and, therefore, unlikely to affect success of restoration efforts. However, most of the traits measured would be expected to be positively correlated based on physiological first principles. Many of the traits included measure different aspects of photosynthesis and would be expected to be positively correlated (e.g. chlorophyll, symbiont counts and ETRmax, and P:R etc). Photosynthesis is also known to be positively correlated with coral calcification (BW, alkalinity). The immune proteins are biochemically entwined because they are inter-conversions of each other. In my view, support for the conclusions that this paper proposes could only come from measurement of a wider range of traits e.g, growth versus survival, linear extension versus protein content, growth versus disease resistance. The rationale for the traits measured that makes a compelling case that at least some of the traits measure processes that aren't directly linked biochemically is required. Apologies to the authors, I know this is likely an annoying comment because you can't change what traits you measured at this stage. However, there needs to be a better explanation of why these traits were chosen, and how testing correlations among these traits specifically can support the conclusions.

Another question that came to mind for me when reading the introduction relates to disase. Given that disease is such a large agent of coral mortality in the Caribbean and Florida specifically, don't we need to look for traits that are positively associated with disease tolerance? I recognise that the immune proteins were likely measured as an indicator of coral capacity to resist disease but would LOVE to see disease tolerance measured more directly. Imagine if we could find a genotype that grew quickly, had high tissue biomass, and was able to resist disease?

A few other minor edits
Table 1 - Temp is higher in the combined treatment. DO changes with temperature. Implications of this?
Line 155 - Should raceway also be tested as a random factor. Unlikely to have a bigger effect than tank BUT should rule this out (or show that including tank alone was statistically supported over including raceway | tank
Line 161 - Just Kenkel et al in text not C. D. Kenkel et al
Line 165 - 'genotype effects by the sum of the variance in all other random factors' - I think tank is the only other random factor right? Is there a source of random variation that could have persisted since collection in the field? Position in the nursery or something?
Line 310 - needs clarification about the 'three comparisions essential for detecting tradeoffs. I had understood that it was the correlation across traits measured in the 12 genotypes that was

important?

Decision letter (RSPB-2021-0923.R0)

05-Jul-2021

Dear Dr Muller:

Your manuscript has now been peer reviewed and the reviews have been assessed by an Associate Editor. The reviewers' comments (not including confidential comments to the Editor) and the comments from the Associate Editor are included at the end of this email for your reference. As you will see, the reviewers and the Editors have raised some concerns with your manuscript and we would like to invite you to revise your manuscript to address them.

We do not allow multiple rounds of revision so we urge you to make every effort to fully address all of the comments at this stage. If deemed necessary by the Associate Editor, your manuscript will be sent back to one or more of the original reviewers for assessment. If the original reviewers are not available we may invite new reviewers. Please note that we cannot guarantee eventual acceptance of your manuscript at this stage.

To submit your revision please log into http://mc.manuscriptcentral.com/prsb and enter your Author Centre, where you will find your manuscript title listed under "Manuscripts with Decisions." Under "Actions", click on "Create a Revision". Your manuscript number has been appended to denote a revision.

When submitting your revision please upload a file under "Response to Referees" - in the "File Upload" section. This should document, point by point, how you have responded to the reviewers' and Editors' comments, and the adjustments you have made to the manuscript. We require a copy of the manuscript with revisions made since the previous version marked as 'tracked changes' to be included in the 'response to referees' document.

Your main manuscript should be submitted as a text file (doc, txt, rtf or tex), not a PDF. Your figures should be submitted as separate files and not included within the main manuscript file.

When revising your manuscript you should also ensure that it adheres to our editorial policies (https://royalsociety.org/journals/ethics-policies/). You should pay particular attention to the following:

Research ethics:
If your study contains research on humans please ensure that you detail in the methods section whether you obtained ethical approval from your local research ethics committee and gained informed consent to participate from each of the participants.

Use of animals and field studies:
If your study uses animals please include details in the methods section of any approval and licences given to carry out the study and include full details of how animal welfare standards were ensured. Field studies should be conducted in accordance with local legislation; please include details of the appropriate permission and licences that you obtained to carry out the field work.

Data accessibility and data citation:
It is a condition of publication that you make available the data and research materials supporting the results in the article. Please see our Data Sharing Policies

(https://royalsociety.org/journals/authors/author-guidelines/#data). Datasets should be deposited in an appropriate publicly available repository and details of the associated accession number, link or DOI to the datasets must be included in the Data Accessibility section of the article (https://royalsociety.org/journals/ethics-policies/data-sharing-mining/). Reference(s) to datasets should also be included in the reference list of the article with DOIs (where available).

In order to ensure effective and robust dissemination and appropriate credit to authors the dataset(s) used should also be fully cited and listed in the references.

If you wish to submit your data to Dryad (http://datadryad.org/) and have not already done so you can submit your data via this link http://datadryad.org/submit?journalID=RSPB&manu=(Document not available), which will take you to your unique entry in the Dryad repository.

If you have already submitted your data to dryad you can make any necessary revisions to your dataset by following the above link.

For more information please see our open data policy http://royalsocietypublishing.org/data-sharing.

Electronic supplementary material:
All supplementary materials accompanying an accepted article will be treated as in their final form. They will be published alongside the paper on the journal website and posted on the online figshare repository. Files on figshare will be made available approximately one week before the accompanying article so that the supplementary material can be attributed a unique DOI. Please try to submit all supplementary material as a single file.

Online supplementary material will also carry the title and description provided during submission, so please ensure these are accurate and informative. Note that the Royal Society will not edit or typeset supplementary material and it will be hosted as provided. Please ensure that the supplementary material includes the paper details (authors, title, journal name, article DOI). Your article DOI will be 10.1098/rspb.[paper ID in form xxxx.xxxx e.g. 10.1098/rspb.2016.0049].

Please submit a copy of your revised paper within three weeks. If we do not hear from you within this time your manuscript will be rejected. If you are unable to meet this deadline please let us know as soon as possible, as we may be able to grant a short extension.

Thank you for submitting your manuscript to Proceedings B; we look forward to receiving your revision. If you have any questions at all, please do not hesitate to get in touch.

Best wishes,
Dr Locke Rowe
mailto: proceedingsb@royalsociety.org

Associate Editor
Board Member: 1
Comments to Author:
Both reviewers were positive about this ms on the responses of a branching coral to heat and ocean acidification. They do however raise some questions about the methodology and interpretations which will need to be addressed by the authors. These relate especially to the interpretation of the synergistic effect between warming and acidification (R1), and to the likely positive correlations between traits that were used to assess tradeoffs (R2). In additional comments, both reviewers also requested that access to the supporting data be made clearer or simpler than the current links to the NSF database.

Reviewer(s)' Comments to Author:
Referee: 1
Comments to the Author(s)
This study tracks the performance of 12 coral genets that are currently used in restoration over two months in elevated temp and pCO2 lab treatments. Unlike many studies of this type, the authors track a large number of physiological trait responses (11 parameters, related to host, symbionts, and holobiont) to the climate change stressors with a relatively large number of genets. The results thus underscore that different traits show both different patterns of response to the stressors and different patterns of genotypic responsiveness (hence expected adaptability). The apparent lack of tradeoffs is particularly encouraging, and more convincing in this paper than others I am familiar with that have tried to examine this question. The paper is quite well-written and the data is clearly presented. I have primarily minor suggestions for clarification (and one slight correction).
My one substantive comment relates to the treatment conditions and the conclusions of widespread synergistic effects. However, it appears from Table 1 that the treatment levels in the Combined treatment were higher (and for temp considerably higher and more variable) than in the respective single stressor treatments. For temp this was 1.4oC higher (and it seems that a 1.2 degree standard error means it got substantially above 32.5 in this combined treatment for at least a portion of the time) which seems substantial to me. Could this increment in heat stress not account for some of the larger effect sizes that you attribute as synergism? This synergy of stressors is a major conclusion/discussion point which requires better justification given this apparent discrepancy in the treatment levels.

Minor points:
-Question: is there a reason the authors use the term ‘Florida’s Coral Reef’ rather than the long extablished ‘Florida Reef Tract’?
Ln 50-52: I found this a rather strong statement regarding Florida’s reef currently (i.e. present tense) experiencing effects of acidification impairing growth and skeletal integrity and indeed, none of the cited references pertain to FCR. The Florida-related reference for reef dissolution in the following sentence does not distinguish effects of acidification from the simple fact that Floridas reefs simply have extremely low abundance of calcifying corals!
Ln 62-63: I am also not sure the statement that Floridas ‘iconic reefs’ restoration project is the ‘largest . . planned in the world’. It is massive, but there are certainly similar planning efforts underway (if not as advanced as the Florida effort) for Australia’s GBR no?
Ln 79: consider revision to ‘phenotypes upon which selection can act’
Experimental design description: I know it is common practice to refer to certain treatments levels as ‘ambient’. However, this the description for this paper, it is explicitly clear that the ‘ambient’ temperature treatment is not as it involved shifting from 30.35 at which the corals were collected to 27! If 27 is not the ambient temp, don’t call it that. Just call it 27.5! I am not sure what the actual ‘ambient’ pCO2 level is . . . but please consider if the ‘control’ pCO2 level you are using is really ‘ambient’
Ln122-124: It would be nice to include a sentence regarding how pCO2 was manipulated in this study (presumably by bubbling CO2 into the system water?)
Ln 144-146: I would have appreciated a sentence explaining why the PPO, PO, and POX are relevant or of interest.
Ln 171-175: My interpretation of this correlation analysis is that there is an unstated assumption that all of the parameters/traits measured are ‘beneficial’ in some sense, such that higher levels always represent a better status for the organism. Am I off base here? I am not completely convinced that this assumption makes sense (e.g., is higher symbiodinium cell density or higher protein content always better?). I guess I think the rationale for this analysis could use a better explanation (as I feel I am not understanding the logic).
Ln 199: Should this word be ‘indicators’?
Ln 225 and Ln 260: these instances should be effect (not affect)
Ln 233: should be ‘combined’
Ln 268: reconsider the term ‘negative decline’ – this sounds like a double negative that would be positive

Ln 350: delete one more
Figs 2-4: I like the way these figs are presented (very clear). However, I think the legend for Fig 4 should say 'symbionts' rather than 'host'
General for all figs: I imagine this is just an artifact of the proof (i.e. not the authors' doing), but the fonts are WAY too small on all the figure renderings in the review copy.

Referee: 2
Comments to the Author(s)
I like this paper. It is novel and timely, and the experimental design and data analysis are rigorous and robust. The papers is clear and concise, very well written overall.

My issue with the paper is a conceptual one with regard to the choice of traits measured and tested. The conclusions of the paper are that tradeoffs between traits are not likely to impact survival of coral fragments and, therefore, unlikely to affect success of restoration efforts. However, most of the traits measured would be expected to be positively correlated based on physiological first principles. Many of the traits included measure different aspects of photosynthesis and would be expected to be positively correlated (e.g. chlorophyll, symbiont counts and ETRmax, and P:R etc). Photosynthesis is also known to be positively correlated with coral calcification (BW, alkalinity). The immune proteins are biochemically entwined because they are inter-conversions of each other. In my view, support for the conclusions that this paper proposes could only come from measurement of a wider range of traits e.g, growth versus survival, linear extension versus protein content, growth versus disease resistance. The rationale for the traits measured that makes a compelling case that at least some of the traits measure processes that aren't directly linked biochemically is required. Apologies to the authors, I know this is likely an annoying comment because you can't change what traits you measured at this stage. However, there needs to be a better explanation of why these traits were chosen, and how testing correlations among these traits specifically can support the conclusions.

Another question that came to mind for me when reading the introduction relates to disase. Given that disease is such a large agent of coral mortality in the Caribbean and Florida specifically, don't we need to look for traits that are positively associated with disease tolerance? I recognise that the immune proteins were likely measured as an indicator of coral capacity to resist disease but would LOVE to see disease tolerance measured more directly. Imagine if we could find a genotype that grew quickly, had high tissue biomass, and was able to resist disease?

A few other minor edits
Table 1 - Temp is higher in the combined treatment. DO changes with temperature. Implications of this?
Line 155 - Should raceway also be tested as a random factor. Unlikely to have a bigger effect than tank BUT should rule this out (or show that including tank alone was statistically supported over including raceway|tank
Line 161 - Just Kenkel et al in text not C. D. Kenkel et al
Line 165 - 'genotype effects by the sum of the variance in all other random factors' - I think tank is the only other random factor right? Is there a source of random variation that could have persisted since collection in the field? Position in the nursery or something?
Line 310 - needs clarification about the 'three comparisions essential for detecting tradeoffs. I had understood that it was the correlation across traits measured in the 12 genotypes that was important?

Author's Response to Decision Letter for (RSPB-2021-0923.R0)

See Appendix A.

RSPB-2021-0923.R1 (Revision)

Review form: Reviewer 1

Recommendation
Accept as is

Scientific importance: Is the manuscript an original and important contribution to its field?
Excellent

General interest: Is the paper of sufficient general interest?
Good

Quality of the paper: Is the overall quality of the paper suitable?
Excellent

Is the length of the paper justified?
Yes

Should the paper be seen by a specialist statistical reviewer?
No

Do you have any concerns about statistical analyses in this paper? If so, please specify them explicitly in your report.
No

It is a condition of publication that authors make their supporting data, code and materials available - either as supplementary material or hosted in an external repository. Please rate, if applicable, the supporting data on the following criteria.

> **Is it accessible?**
> Yes
>
> **Is it clear?**
> Yes
>
> **Is it adequate?**
> Yes

Do you have any ethical concerns with this paper?
No

Comments to the Author
Thank you for revisions. I am glad the discrepancy in the treatment conditions was attributable to typos so in the end the experimental design is not compromised.

Review form: Reviewer 2

Recommendation
Accept as is

Scientific importance: Is the manuscript an original and important contribution to its field?
Excellent

General interest: Is the paper of sufficient general interest?
Good

Quality of the paper: Is the overall quality of the paper suitable?
Good

Is the length of the paper justified?
Yes

Should the paper be seen by a specialist statistical reviewer?
No

Do you have any concerns about statistical analyses in this paper? If so, please specify them explicitly in your report.
No

It is a condition of publication that authors make their supporting data, code and materials available - either as supplementary material or hosted in an external repository. Please rate, if applicable, the supporting data on the following criteria.

> **Is it accessible?**
> Yes
>
> **Is it clear?**
> Yes
>
> **Is it adequate?**
> Yes

Do you have any ethical concerns with this paper?
No

Comments to the Author
Thanks for your thoughtful consideration of the points I raised in my review. I am happy to hear that you are doing additional studies integrating the traits measured here with other ecologically-relevant traits. I appreciate the inclusion of the among-trait correlation matrices in the supplementary information and am happy to recommend the paper be published in Proc B.

Looking forward to seeing the next installments as well.

Decision letter (RSPB-2021-0923.R1)

21-Sep-2021

Dear Dr Muller

I am pleased to inform you that your manuscript entitled "Heritable variation and lack of tradeoffs suggest adaptive capacity in Acropora cervicornis despite negative synergism under climate change scenarios" has been accepted for publication in Proceedings B.

You can expect to receive a proof of your article from our Production office in due course, please check your spam filter if you do not receive it. PLEASE NOTE: you will be given the exact page length of your paper which may be different from the estimation from Editorial and you may be asked to reduce your paper if it goes over the 10 page limit.

If you are likely to be away from e-mail contact please let us know. Due to rapid publication and an extremely tight schedule, if comments are not received, we may publish the paper as it stands.

If you have any queries regarding the production of your final article or the publication date please contact procb_proofs@royalsociety.org

Data Accessibility section
Please remember to make any data sets live prior to publication, and update any links as needed when you receive a proof to check. It is good practice to also add data sets to your reference list.

Open Access
You are invited to opt for Open Access, making your freely available to all as soon as it is ready for publication under a CCBY licence. Our article processing charge for Open Access is £1700. Corresponding authors from member institutions (http://royalsocietypublishing.org/site/librarians/allmembers.xhtml) receive a 25% discount to these charges. For more information please visit http://royalsocietypublishing.org/open-access.

Your article has been estimated as being 10 pages long. Our Production Office will be able to confirm the exact length at proof stage.

Paper charges
An e-mail request for payment of any related charges will be sent out after proof stage (within approximately 2-6 weeks). The preferred payment method is by credit card; however, other payment options are available

Electronic supplementary material:
All supplementary materials accompanying an accepted article will be treated as in their final form. They will be published alongside the paper on the journal website and posted on the online figshare repository. Files on figshare will be made available approximately one week before the accompanying article so that the supplementary material can be attributed a unique DOI.

Thank you for your fine contribution. On behalf of the Editors of the Proceedings B, we look forward to your continued contributions to the Journal.

Sincerely,
Dr Locke Rowe
Editor, Proceedings B
mailto: proceedingsb@royalsociety.org

Associate Editor:
Board Member: 1
Comments to Author:
The authors made appropriate revisions to the manuscript and supporting material in the light of the review comments, and these were appreciated by both reviewers.

Appendix A

Associate Editor
Board Member: 1
Comments to Author:
Both reviewers were positive about this ms on the responses of a branching coral to heat and ocean acidification. They do however raise some questions about the methodology and interpretations which will need to be addressed by the authors. These relate especially to the interpretation of the synergistic effect between warming and acidification (R1), and to the likely positive correlations between traits that were used to assess tradeoffs (R2). In additional comments, both reviewers also requested that access to the supporting data be made clearer or simpler than the current links to the NSF database.

RESPONSE: Thank you very much to our Associate Editor and both reviewers. We appreciate the thorough review and helpful feedback. We have addressed the three major recommendations by the following:

Combined Treatments conditions and synergistic response detected: We have addressed the interpretation of the synergistic response of the 'combined treatment' in two ways 1. having conducted another qa/qc of the temperature data we found several instances where the salinity was entered into the spreadsheet instead of the appropriate temperature values (adjusting this made the SE similar among treatments), and 2. Added statements into the Discussion regarding the challenges of a complex experimental design with system limitations and the interplay of many water quality parameters making it difficult to keep each parameter exactly the same among multiple treatment scenarios.

Positive Correlations: Yes, we understand and discussed this among co-authors prior to submission. However, our analyses show that even some metrics that, in theory, may correlate have differential responses associated with these treatment scenarios. Therefore, in the revision we have included a correlation matrix analysis, identified if and where any correlative traits occurred, and provided an additional figure of this analysis within the supplemental document. This approach allowed us to still show the variations among metrics measured while being transparent about potential correlations among these traits.

Data Repository: We have uploaded our data in Dryad, for easier and transparent access. Thank you for the suggestion.

Muller, Erinn et al. (2021), Data from: Heritable variation and lack of tradeoffs suggest adaptive capacity in Acropora cervicornis despite negative synergism under climate change scenarios, Dryad, Dataset, https://doi.org/10.5061/dryad.mcvdnck1w (this link we be activated upon publication)

Direct Link: https://datadryad.org/stash/share/TVCbEMrPAt-7dW788b2tOxNw5QQOV1C_t8qAk4FbXn8

Reviewer(s)' Comments to Author:

Referee: 1

Comments to the Author(s)

This study tracks the performance of 12 coral genets that are currently used in restoration over two months in elevated temp and pCO2 lab treatments. Unlike many studies of this type, the authors track a large number of physiological trait responses (11 parameters, related to host, symbionts, and holobiont) to the climate change stressors with a relatively large number of genets. The results thus underscore that different traits show both different patterns of response to the stressors and different patterns of genotypic responsiveness (hence expected adaptability). The apparent lack of tradeoffs is particularly encouraging, and more convincing in this paper than others I am familiar with that have tried to examine this question. The paper is quite well-written and the data is clearly presented. I have primarily minor suggestions for clarification (and one slight correction).

RESPONSE: Thank you very much for reviewing our manuscript and we appreciate the positive feedback. We are so grateful for your suggestions and feel the paper is much improved because of your contributions. It was a TON of work and we are looking forward to contributing to the field should it be deemed accepted in Proc B.

My one substantive comment relates to the treatment conditions and the conclusions of widespread synergistic effects. However, it appears from Table 1 that the treatment levels in the Combined treatment were higher (and for temp considerably higher and more variable) than in the respective single stressor treatments. For temp this was 1.4oC higher (and it seems that a 1.2 degree standard error means it got substantially above 32.5 in this combined treatment for at least a portion of the time) which seems substantial to me. Could this increment in heat stress not account for some of the larger effect sizes that you attribute as synergism? This synergy of stressors is a major conclusion/discussion point which requires better justification given this apparent discrepancy in the treatment levels.

RESPONSE: Thank you very much for pointing out this issue with the temperature averages and standard errors. We conducted a thorough qa/qc for all 938 data points and realized that there were several data entry issues within the data set used to compile Table 1. The updated table now shows similar levels of variation in temperature among the treatments and only 0.1 C differences in averages among target values. We also add in text recognizing the differences in DO among treatments as it is inherently lower in the high temperature treatments due to reduced solubility of gases as temperature increases. Specifically, we added in the following text:

“Treatments within the present study, although constantly maintained, differed from target values due to the complexity associated with the experimental design and system limitations. Additionally, the interplay between water temperature and dissolved gases (negative association with dissolved oxygen and positive association with $p\text{CO}_2$) prevented maintaining the same water conditions within the four treatment scenarios. These methodological limitations may have influenced phenotypic responses associated with treatment exposure within the present study.

Treatment	Temperature (°C)	Salinity (ppt)	DO (mg/L)	Flow rates (cm s^{-1})	PAR (μmol photons m^{-2} s^{-1})	pH_T	TA (μmol kg^{-1} SW)	pCO_2 (μatm)	[HCO_3^-] (μmol kg^{-1} SW)	[CO_3^{2-}] (μmol kg^{-1} SW)	Ωar
Control	27.1±0.05	36.5±0.1	7.2±0.03	8.8±4.8	273±9	7.85±0.01	2168±19	704±62	1777±24	143±6	2.25±0.02
High pCO_2	27.0±0.02	36.5±0.1	7.1±0.03	10.8±7.1	273±9	7.60±0.02	2179±17	1225±98	1910±23	97±8	1.54±0.3
High Temperature	31.0±0.04	36.5±0.1	6.9±0.04	11.4±5.7	273±9	7.8±0.01	2198±19	798±62	1793±21	142±6	2.27±0.2
Combined											1.51±0.03

Minor points:
-Question: is there a reason the authors use the term 'Florida's Coral Reef' rather than the long extablished 'Florida Reef Tract'?

RESPONSE: The state managers of Florida (Florida Fish and Wildlife and Florida Department of Protection) have 'rebranded' the Florida Reef Tract as Florida's Coral Reef in order to acquire the sense of ownership from the public, and to further gain attention to the plight of the ecosystem. They have requested that those of us publishing and communicating our research associated with the region use the newly branded name.

Ln 50-52: I found this a rather strong statement regarding Florida's reef currently (i.e. present tense) experiencing effects of acidification impairing growth and skeletal integrity and indeed, none of the cited references pertain to FCR. The Florida-related reference for reef dissolution in the following sentence does not distinguish effects of acidification from the simple fact that Floridas reefs simply have extremely low abundance of calcifying corals!

RESPONSE: Thank you for pointing this out. We agree with your recommendation and have adjusted the sentence, added specific references associated with the Florida Keys, and adjusted the text to read: **"**FCR also experiences a combination of ocean and coastal acidification processes (Muehllehner et al., 2016; Enochs et al., 2019; Meléndez et al., 2020), which can impair coral growth and skeletal integrity (Albright & Langdon, 2011; Anthony, Kline, Diaz-Pulido, Dove, & Hoegh-Guldberg, 2008; Cohen, McCorkle, De Putron, Gaetani, & Rose, 2009; Fine & Tchernov, 2007; Krief et al., 2010; Meléndez et al., 2020). Ocean acidification is the global decrease in oceanic pH as a result of the exponential increase of atmospheric carbon dioxide since the Industrial Revolution, which in turns leads to increases of dissolved CO_2 within the oceans [24,25]. Ocean acidification on FCR acts in conjunction with coastal acidification, which results from microbial degradation of organic matter from freshwater inputs and excess nutrient run-off in coastal zones [26,27]. Indeed, Muehllehner, Langdon, Venti, & Kadko (2016) showed that reefs within the Florida Keys had a net dissolution rate while Moses et al. (2008) determined that those reefs

31.1±0.05 36.4±0.1 6.6±0.03 10.6±4.7 273±9 7.54±0.01 2191±17 1412±90 194

are a net sink for CO_2, and Gledhill et al. (2008) showed a decrease in aragonite saturation state, suggesting acidification may already be affecting coral reefs in this region.

Ln 62-63: I am also not sure the statement that Floridas 'iconic reefs' restoration project is the 'largest . . planned in the world'. It is massive, but there are certainly similar planning efforts underway (if not as advanced as the Florida effort) for Australia's GBR no?

RESPONSE: This point is well taken and we have adjusted the statement to say "one of the largest coordinated coral restoration efforts…"

Ln 79: consider revision to 'phenotypes upon which selection can act'

RESPONSE: Great suggestion. Thank you. We have updated the text as recommended.

Experimental design description: I know it is common practice to refer to certain treatments levels as 'ambient'. However, this the description for this paper, it is explicitly clear that the 'ambient' temperature treatment is not as it involved shifting from 30.35 at which the corals were collected to 27! If 27 is not the ambient temp, don't call it that. Just call it 27.5! I am not sure what the actual 'ambient' pCO2 level is . . . but please consider if the 'control' pCO2 level you are using is really 'ambient'

RESPONSE: Great point. We have adjusted the text to identify the treatment values and define what each treatment represents with values rather than treatment names alone. We provided additional context to the section as well to better represent actual treatment conditions and limitations associated with hitting ideal targets. The edits can be found from Line 141 to 145.

Ln122-124: It would be nice to include a sentence regarding how pCO2 was manipulated in this study (presumably by bubbling CO2 into the system water?)

RESPONSE: Thank you for pointing out this omission. We have added the following: "The high pCO_2 treatments were decreased by 0.1 pH units per day (over the span of 4 days) by bubbling CO_2 within the source water header tank"

Ln 144-146: I would have appreciated a sentence explaining why the PPO, PO, and POX are relevant or of interest.

RESPONSE: Great suggestion. We have added the following sentences to better explain our use of these immune metrics " The melanin synthesis cascade, a key component of innate immunity in invertebrates, is activated by the proteolysis of PPO to form active PO (Mydlarz and Palmer, 2011). Reactive oxygen intermediates are generated as part of this cascade, which can be cytotoxic to the pathogens as well as to the coral host, consequently, antioxidants such as POX are formed, presumably as a means of minimizing cytotoxicity to host cells (Mydlarz and Palmer, 2011). *Acropora cervicornis* genets are known to vary in disease susceptibility (Muller et al. 2018) and immune competence, therefore, the activity of these three enzymes were quantified as a proxy of host immune function."

Ln 171-175: My interpretation of this correlation analysis is that there is an unstated assumption that all of the parameters/traits measured are 'beneficial' in some sense, such that higher levels always represent a better status for the organism. Am I off base here? I am not completely convinced that this assumption makes sense (e.g., is higher symbiodinium cell density or higher protein content always better?). I guess I think the rationale for this analysis could use a better explanation (as I feel I am not understanding the logic).

RESPONSE: Thank you for your suggestion, here. We have added clarifying text associated with the methodology section, which better explains why we measured each trait and the implications. Text for this is associated with lines 177 to 206 within the revised manuscript.

We also explicitly added in the following statement to assist with the data interpretation "In all cases, corals within the Control treatment were considered at a standard level of performance. Because potentially 'beneficial' metrics, such as significant growth or higher immune function, under treatment scenarios were not observed, deviations from the Control metrics were assumed to be physiological negative responses."

Ln 199: Should this word be 'indicators'?

RESPONSE: Yes, thank you. We have completed this edit.

Ln 225 and Ln 260: these instances should be effect (not affect)

RESPONSE: Yes, thank you. We have completed these edits.

Ln 233: should be 'combined'

RESPONSE: Thank you. We have completed this edit.

Ln 268: reconsider the term 'negative decline' – this sounds like a double negative that would be positive

RESPONSE: Great suggestion. We have change the text to say "...significant reduction in Yield"

Ln 350: delete one more

RESPONSE: Thank you. We have completed this edit.

Figs 2-4: I like the way these figs are presented (very clear). However, I think the legend for Fig 4 should say 'symbionts' rather than 'host'

RESPONSE: Great catch. Thank you. This has been changed in the figure legend.

General for all figs: I imagine this is just an artifact of the proof (i.e. not the authors' doing), but the fonts are WAY too small on all the figure renderings in the review copy.

RESPONSE: Thank you for suggesting this change. There were some issues with font changes associated with the saving process in R. These have now been optimized for easier reading.

Referee: 2

Comments to the Author(s)
I like this paper. It is novel and timely, and the experimental design and data analysis are rigorous and robust. The papers is clear and concise, very well written overall.

My issue with the paper is a conceptual one with regard to the choice of traits measured and tested. The conclusions of the paper are that tradeoffs between traits are not likely to impact survival of coral fragments and, therefore, unlikely to affect success of restoration efforts. However, most of the traits measured would be expected to be positively correlated based on physiological first principles. Many of the traits included measure different aspects of photosynthesis and would be expected to be positively correlated (e.g. chlorophyll, symbiont counts and ETRmax, and P:R etc). Photosynthesis is also known to be positively correlated with coral calcification (BW, alkalinity). The immune proteins are biochemically entwined because they are inter-conversions of each other. In my view, support for the conclusions that this paper proposes could only come from measurement of a wider range of traits e.g, growth versus survival, linear extension versus protein content, growth versus disease resistance. The rationale for the

traits measured that makes a compelling case that at least some of the traits measure processes that aren't directly linked biochemically is required. Apologies to the authors, I know this is likely an annoying comment because you can't change what traits you measured at this stage. However, there needs to be a better explanation of why these traits were chosen, and how testing correlations among these traits specifically can support the conclusions.

RESPONSE: Thank you for your thoughtful insight and suggestions. We grappled with this as well, however, our approach quantified multiple metrics that encompass important traits associated with thermal tolerance (Yield, ETRm, Total Chl, Symbiont density), host health and immunity (protein, immune protein assays), physiological status (p:r), and growth (buoyant weight, calcification) – all metrics that are important for characterizing phenotypic responses to stress. Our data suggests that the metric chosen to assess these broader traits can substantially influence the interpretation of the results. For example, buoyant weight and calcification (light and dark) showed different responses to the treatments, with BW showing potential synergism in the combined treatment and calcification showing an antagonistic response within this treatment. Similarly, although PO and PPO responded similarly, POX was more reduced within the high temperature treatment compared to others. These nuances among traits measured are important to include thereby helping guide selecting metrics to measure in future experiments. At this point, I don't think we can identify exactly which traits are the most important to measure. Although we do think it is important to leave these metrics within the paper, we certainly want to be transparent that some metrics are indeed often broadly correlated. We had already completed this cross correlation analysis prior to submission, but removed it due to length requirements. Within this revision we have added the figure back into the paper, but show it within the Supplemental document. For ease of clarity, we have identified which traits are often significantly correlated among each other within the correlation figure within the main document. Interestingly, very few (only two) correlate between components of the holobiont.

We also appreciate your suggestions of comparing growth with important metrics such as survival, protein content, and disease resistance. Some of these comparisons are being assessed in other studies we have completed, but the goal of the present study was to assess metrics by pushing the corals into stressful conditions under an ecologically relevant two month long time frame without causing mortality so that there was tissue for measuring things like protein, zoox counts, total chlorophyll, and immune proteins. Causing mortality through extreme environmental conditions or exposing corals to disease (which causes mortality) would have prevented us from acquiring the appropriate tissue for these metrics. However, as I mentioned, we do have studies completed that are trying to address your important recommendations.

Another question that came to mind for me when reading the introduction relates to disease. Given that disease is such a large agent of coral mortality in the Caribbean and Florida specifically, don't we need to look for traits that are positively associated with disease tolerance? I recognise that the immune proteins were likely measured as an indicator of coral capacity to resist disease but would LOVE to see disease tolerance measured more directly. Imagine if we could find a genotype that grew quickly, had high tissue biomass, and was able to resist disease?

RESPONSE: Yes, this is exactly where we are headed. We originally planned on incorporating specific genotype ‘ranks’ of resistance within the paper, but it was too much to digest in a single paper. Our plan is to follow up with a specific paper presenting specific genotypic responses collected in the present study and a repeated study we conducted in 2017 using the same treatments and the same genets. We will compare the results of the genotype ranks of the two studies and also include the disease resistance data published in 2018 (Muller et al. eLife) of the same genets. We also have a separate study comparing fecundity of disease resistant and susceptible genets currently in prep.

_

A few other minor edits
Table 1 - Temp is higher in the combined treatment. DO changes with temperature. Implications of this?

RESPONSE: As noted above, there was data entry issues with the spreadsheet used for Table 1, which have now been rectified through an additional qa/qc process. We have also included the fact that several metrics change with temperature (like DO and pCO_2) within the Discussion of the paper. The edits can be found from Line 398 to 406.

Line 155 - Should raceway also be tested as a random factor. Unlikely to have a bigger effect than tank BUT should rule this out (or show that including tank alone was statistically supported over including raceway|tank

RESPONSE: Unfortunately, we could not replicate raceways for each treatment due to system limitations. Because of this, the analyses cannot be conducted by including raceway as a random factor within the model.

Line 161 - Just Kenkel et al in text not C. D. Kenkel et al

RESPONSE: Thank you. We have completed this edit.

Line 165 - 'genotype effects by the sum of the variance in all other random factors' - I think tank is the only other random factor right? Is there a source of random variation that could have persisted since collection in the field? Position in the nursery or something?

RESPONSE: Yes, this is a good point and the more we learn about the plasticity of *A. cervicornis* genets, the more I think position within a tree or within the nursery could be important. However, the team simply haphazardly chose fragments from nursery trees that were of appropriate size for experimentation (5 cm fragments on the branch tips with one apical polyp) and the position of the fragments within a tree

or nursery was not recorded. In reviewing the text we adjusted the methods to read “Twenty fragments with one apical polyp were collected haphazardly throughout the nursery from each of 12 genotypes …” in order to better reflect the methodology.

Line 310 - needs clarification about the 'three comparisions essential for detecting tradeoffs. I had understood that it was the correlation across traits measured in the 12 genotypes that was important?

RESPONSE: Yes, you are certainly correct. The interpretation we were trying to express is in relation to future ocean conditions, which obviously wasn’t communicated well within the texts. We have added the text to clarify (bold shows added text): “Significant positive associations between three key comparisons essential for detecting tradeoffs or conferred broad-spectrum resistance within future ocean conditions (High Temperature vs. High pCO_2, High Temperature vs. Combined, and High pCO_2 vs. Combined)
